# The Relationship of Albuminuria to the Life Insurance Company

**Published:** 1904-09

**Authors:** W. E. Wilmerding

**Affiliations:** Atlanta, Ga.


### THE RELATIONSHIP OF ALBUMINURIA TO THE LIFE INSURANCE COMPANY.

BY W. E. WILMERDING, M.D.,
ATLANTA, GA.

That the presence of albumin in the urine is of prime importance to the insurance company is readily seen, when the frequency of this condition is remembered, about 33 per cent. of all urine showing some albumin, delicate tests being used. Should a company reject all applicants having even a trace of albumin then that company would lose a large volume of business the greater part of which would be perfectly good, while at the same time the protection afforded by insurance is denied to many justly entitled to it. On the other hand the indiscriminate acceptance of risks of this class would certainly show a larger death-rate, thereby working a hardship upon the company and indirectly upon the policy-holders in decreased dividends, etc. It is between these two extremes that the companies should steer, and practically all companies take this view of the matter, requiring additional examinations of the urine to be made for a period of from one to eight or ten weeks, the length of time varying with the different ideas of the various medical directors. It is of course understood that when the albuminuria can be shown to be due to a pathological change in the kidneys the applicant is at once rejected.

A word may be said as to the etiology of albuminuria, though only a word, as this subject has been thoroughly covered by the gentlemen preceding me. Nephritis in its various forms of course occurs to us first and need not be further discussed; postural albuminuria, as first demonstrated by Dr. Stirling, is of great interest, he having conclusively shown that in many cases of albuminuria the albumin entirely disappears while the party is in a recumbent posture, to again be found when the upright position is resumed. Again this condition may be provoked by such adventitious causes as exposure to cold, prolonged or violent muscular exertion, alcoholism, diet, poisoning by lead, phosphorus, etc. Among causes bearing more directly upon the genito-urinary tract may be mentioned hematuria, gonorrhea, pyuria, cystitis, seminal fluid, enlarged prostate and in the female vaginal discharge, leucorrhea, and menstruation.

Leube, Furbringer, Senator and Posner have all shown that even a healthy kidney may excrete a slight quantity of albumin, while on the other hand A. K. Stone, Pye Smith, Washburn and Millard hold to just the opposite view, so that it is quite evident that there is a decided difference of opinion as to whether albumin can be a physiological secretion. However, I believe that the affirmative have the larger following.

To quote from a statement of a medical director of one of the old-line companies: "If on examining the specimen which has been voided just after the patient gets up in the morning, the analysis is negative for albumin and casts, but as the day proceeds, there appears some albumin which gradually disappears as the evening approaches, and finally disappears at bedtime, such cases are insurable if they have a good family and personal record. In all cases of intermittent albuminuria it is important to note before accepting the risk, to find out the amount of urine that he is passing daily and noting if it is in excess of daily amount passed by a normal individual. In a twenty-four-hour specimen, which has been carefully collected and measured, a sample must be taken and the amount of urea estimated normal, the amount being 30 grammes per day, with no history of frequent micturition and with the urine of specific gravity."

This rule is certainly not carried out to the letter, for most companies are satisfied if four or five urinalyses are made at intervals of a few days and during the tide of active digestion, with a microscopical of the last specimen. No albumin being found in any of these tests, the arteries being soft and yielding, and the family and personal record good, then the risk is favorably acted upon and the policy issued.

